# Dengue in Dhaka, Bangladesh: Hospital-based cross-sectional KAP assessment at Dhaka North and Dhaka South City Corporation area

**DOI:** 10.1371/journal.pone.0249135

**Published:** 2021-03-30

**Authors:** Tanvir Abir, O’mezie Ekwudu, Nazmul Ahsan Kalimullah, Dewan Muhammad Nur-A Yazdani, Abdullah Al Mamun, Palash Basak, Uchechukwu Levi Osuagwu, P. Yukthamarani Permarupan, Abdul Hasnat Milton, Shamim Hyder Talukder, Kingsley E. Agho

**Affiliations:** 1 College of Business Administration, International University of Business, Agriculture and Technology, Dhaka, Bangladesh; 2 Institute of Health and Biomedical Innovation, Queensland University of Technology, Brisbane, Queensland, Australia; 3 Begum Rokeya University, Rangpur, Bangladesh; 4 Faculty of Business and Management, UCSI University, Kuala Lumpur, Malaysia; 5 School of Environment and Life Sciences (Environmental Science and Management), University of Newcastle, Callaghan, Australia; 6 Diabetes, Obesity, and Translational Research Unit (DOMTRU), School of Medicine, Western Sydney University, Sydney, Australia; 7 Faculty of Entrepreneurship and Business, Universiti Malaysia Kelantan, Kota Bharu, Malaysia; 8 Epidemiology Resource Centre, Newcastle, Australia; 9 Eminence Associates for Social Development, Bangladesh; 10 School of Health Sciences, Western Sydney University, Sydney, Australia; University of Maryland, UNITED STATES

## Abstract

Dengue, the most important mosquito-borne viral disease of humans is a recurring global health problem. In Bangladesh, dengue outbreaks are on the increase despite the efforts of government and it is not clear what the understanding of the general Dhaka population towards dengue fever is. Knowledge, attitude and practice (KAP) studies are essential guides in public health interventions. Hence, using KAP, this study aims to assess patient-perspectives with regards to factors associated with dengue, as well as investigate the associated factors between the two corporations in Dhaka. A Hospital-based cross-sectional study of 242 fever patients from two city-corporations in Dhaka (Dhaka North City Corporations, DNCC (n = 91, 37.6%) and Dhaka South City Corporation, DSCC (n = 151, 62.4%) was conducted using pre-tested KAP items. Wilcoxon’s Rank Sum was used to determine the KAP by DNCC, DSCC and both corporations and multivariate Poisson regression analyses. The two corporations were analysed separately due to the differences in income distribution, concentration of slums, hospitals and clinics. The study found that more than half of the study population were knowledgeable about dengue (mean percentage scores was 52%), possess an appropriate and acceptable attitude towards the disease (69.2%), and about two thirds of the respondents (71.4%) engaged in practices towards its prevention. After adjusting for the potential cofounders, the factors associated with KAP about dengue fever varied between DNCC and DSCC; with duration of residency and use of mosquito nets were associated with knowledge in the north while income class and age were associated with knowledge and attitude in the south. In the pooled analysis (combining both corporations), knowledge of dengue was associated with good practice towards dengue fever among the respondents. The duration of residence in Dhaka (10+ years), not using mosquito nets and length of time spent in the hospital (7+ days) due to dengue, and decreased knowledge (Adjusted coefficient (β) = -0.01, 95%CI: -0.02, -0.01) were associated with attitude towards dengue in DNCC. On the other hand, middle-high income class, age (40+ years) and increased knowledge were associated with practice towards dengue in DSCC (β = 0.02, 95%CI: 0.01, 0.03). Efforts to increase knowledge about dengue fever through education by the administrations of both corporations would benefit from targeting these high-risk groups for a more sustainable outcome.

## Background

Dengue is an important public health problem and the leading mosquito-borne viral disease of humans. The disease is caused by dengue viruses (DENV); four genetically related but antigenically distinct ribonucleic acid (RNA) viruses (DENV serotypes 1–4) [1]. DENV belongs to the *Flaviviridae* family and the most important vectors for viral transmission between humans are blood-feeding females of the mosquito species, *Aedes aegypti* and *Aedes albopictus*. In recent decades, the incidence of dengue has grown dramatically around the world with as many as 390 million dengue infections projected to occur annually, with a quarter of these showing clinical manifestations [2, 3]. The ongoing rise in the number of dengue cases is due, in a large part, to the extensive spread of mosquito vectors, rapid and unregulated urbanization, increased international travel, and the absence of effective interventions. According to the World Health Organization; the Americas, Southeast Asia (mostly Thailand, Indonesia and the Philippines) and the Western Pacific are the regions most affected by dengue. Seventy-five percent of the world’s dengue burden is in Asia and there have been significant increases in the number of areas becoming hyperendemic; harbouring multiple DENV serotypes [4, 5].

In Bangladesh, South Asia, dengue fever was first reported in 1964 but became a disease of public health significance from 2000 onwards [6, 7]. Bangladesh has a lower dengue prevalence than most Southeast Asian states [8], but recently has sustained an upsurge in dengue outbreaks; from 2769 cases in 2017 to 10148 cases in 2018. In 2019, the Directorate General of Health Services (DGHS) [9] recorded 87953 cases with 81 deaths, a 9-fold increase in the incidence rate of dengue from the previous year [5, 10]. Previous studies indicate that the number of dengue cases and deaths are highest in the warmer months from July to November, and that men were twice as likely to become infected than women [11]. Many cases of dengue are misclassified because of the wide spectrum of disease signs and symptoms and lack of effective case definitions [12]. Therefore, it is highly probable that dengue cases may be substantially under-reported in Bangladesh given the weak surveillance of a struggling healthcare system. Bangladesh is exposed to a continual risk of dengue virus importations from surrounding endemic neighbours like India and Myanmar [13, 14]. Dhaka, the capital and most populated city in Bangladesh, has a population (approximately 16 million) had the highest number of dengue cases between 2012 and 2019 [13, 15]. Like many other countries, the first dengue vaccine, CYD-TDV, has not been rolled out in Bangladesh due to the risk of more severe disease manifestations in seronegative individuals and children less than 9 years [16, 17]. Even though it is unsustainable, and at times ineffective, vector control with insecticides remains the mainstay of dengue prevention strategies [18].

Research into knowledge, attitude and practice (KAP) provides information about health-related behaviours while sampling the awareness of a disease in a population; and therefore can play a critical role in the prevention of disease [19–22]. KAP assessments have been widely ascertained as potent tools in public health intervention research [23–25], where information on what participants know, believe and do could be used to promote specific behaviours and conducts that can improve health outcomes. With the rapidly increasing burden of dengue in Bangladesh, and in view of the recent rise in dengue deaths within the most populated city, there is a need for continuous assessment of people’s knowledge, attitude and practice of the residents of Dhaka to devise intervention plans.

Dhaka comprises of Dhaka North City Corporation (DNCC) and Dhaka South City Corporation (DSCC) with the former having a higher density of lower-income communities (>124,000 persons per square kilometre) and slums (1755 vs 1639), and a lower per capita number of hospitals and clinics (193 vs 293) than the latter [26, 27]. A recent quantitative assessment between these corporations suggests different public health policies due to the economic disparity between both corporations [28].

Therefore, this study aimed to assess the knowledge of dengue among fever patients in conjunction with the prevailing attitude and practices associated with the disease across the economic spectrum of the residents of both corporations. Findings of this study will inform intervention strategies for an effective prevention and control of dengue in both corporations, and the wider Bangladesh.

## Methods

### Study design and sampling

The required sample size was calculated by using the statistical formula:

n0=z2p(1−p)d2

Where n_0_ is the sample size, z^2^ was standard value normal distribution at 95% confidence level (1.96), proportion (p) = 0.50 was considered as the average of gender percentage of dengue cases reported in 2019 from previous study [11], d^2^ was acceptable maximum error (5%). The minimum sample size was 196 and after adding 25% non-response rate yield, the total sample size became 242 respondents. Participants were fever patients presenting with dengue or suspected dengue.

### Ethics statement

This study was approved by the Begum Rokeya University Rangpur Human Research Ethics Committee (HREC Approval Number: BRUR/DWRTI/a.n.001).

### Study design and sampling method

**Fig 1:**
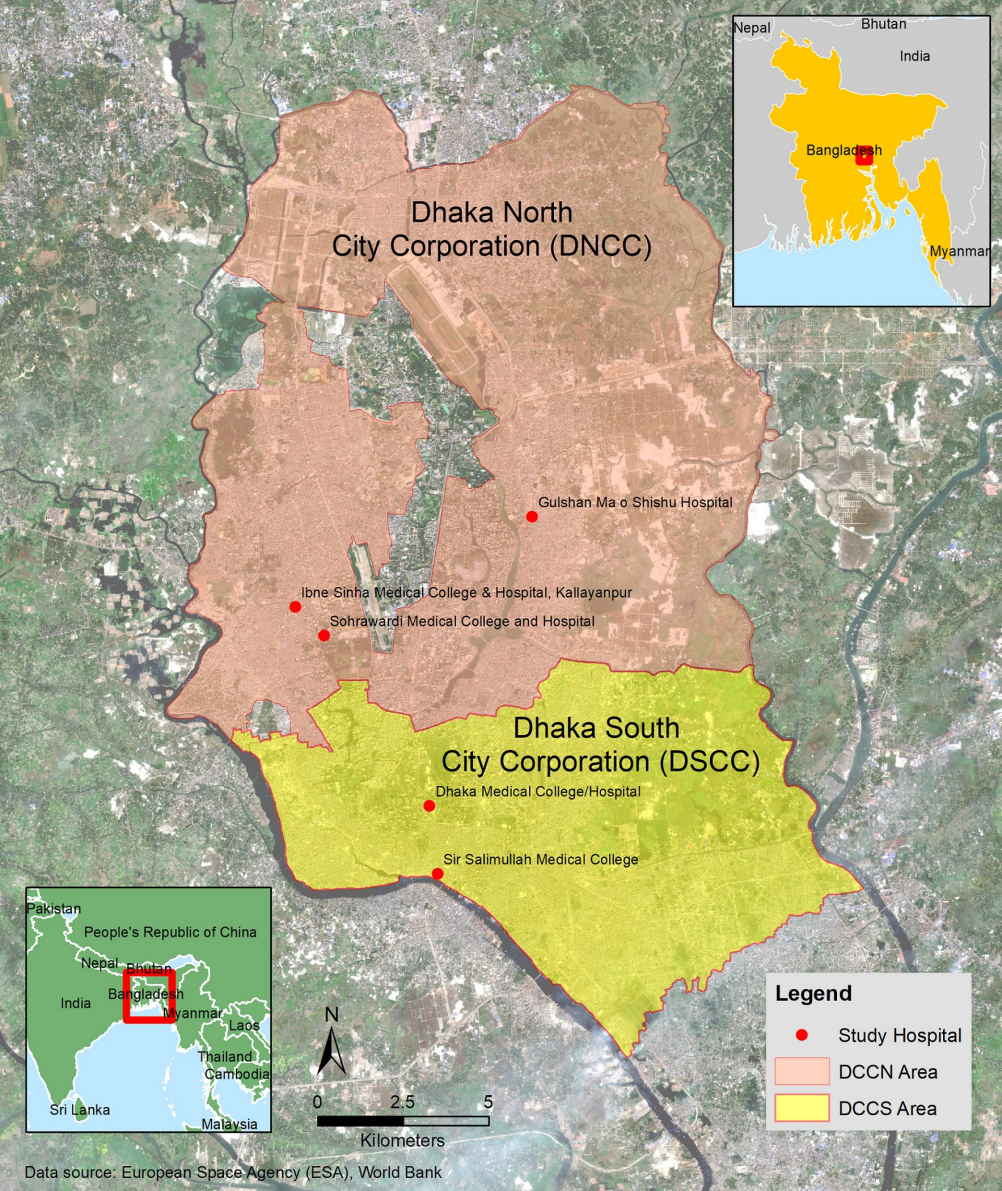
The city corporation boundaries and locations of the surveyed hospitals in DSCC and DNCC.

From August 30 to September 30, 2019, a cross-sectional study was conducted to assess KAP and associated factors related to dengue fever (DF) among a pool of patients who had fever symptoms in the two city-corporations of the Dhaka. Questionnaires (S1 Table) and interviews were administered to ascertain the KAP of 242 dengue fever patients that were randomly selected from multiple hospitals. Fig 1 shows the locations of the study area and hospitals from which survey participants were recruited. In DSCC, there were patients from Dhaka medical college (n = 81) and Sir Salimullah medical college hospitals (n = 70) who had dengue fever and in DNCC, the patients were from Gulshan Ma o Shishu (n = 30), Sohrawardi medical college (n = 31) and Ibne Sinha Medical College, Kallayanpur Branch, Hospitals (n = 30).

### Inclusion criteria and consent

Participants who met the following eligibility criteria were included in the study: (1) Dengue patients in the two Dhaka city-corporations; (2) had dengue fever or dengue symptoms; (3) agreed to participate in this interview by giving written consent; (4) were able to answer the questionnaire. Before interviewing, all respondents were informed of the study details and that the information they gave would be kept confidential and they could stop answering questions at any time.

### Data collection

Data was collected to examine knowledge, attitude, practices regarding DF and its prevention using a semi-structured questionnaire conducted through face-to-face interviews and included questions about epidemiological characteristics. The questionnaire was developed, pre-tested and modified according to the pre-testing results. Reliability of the survey items was computed using Cronbach’s alpha coefficient and resulted in an alpha score of 0.73. Each interview lasted between 10–15 minutes. To ensure the confidentiality of the interview, patients were taken to a private area of the clinic to administer questionnaire, and survey sheets stored in a secure location.

### Dependent variables

There were 27 items on the questionnaire that assessed the respondent’s knowledge about DF and each question used a binary scale. Binary scores for individual questions were summed to give a knowledge score from 0–27. The knowledge items about DF were assessed by asking participants if they could identify clinical symptoms; the means of transmission; the basic means of preventing the propagation of mosquitoes and their larvae in and around their households. The Cronbach’s alpha coefficients measuring internal consistency among the knowledge scores ranged from 0.68 to 0.71, indicating a satisfactory level of reliability [29, 30].

The attitude towards DF measured feelings about the severity of dengue, including the necessity of hospitalization. The attitude questions consisted of 19 items. Each question used a Likert scale with five levels. Each statement was given a score of 5 for “strongly agree”, 4 for “agree”, 3 for “neutral”, 2 for “disagree”, and 1 for “strongly disagree”. The attitude scores were summed to yield an overall attitude score ranging from 0–95 points. The Cronbach’s alpha coefficient for attitude scores ranged from 0.67 to 0.70, indicating acceptable internal consistency [29, 30].

There were 8 items on the questionnaire that assessed dengue prevention practices and items were based on how the participants controlled mosquitoes and larvae in their house, and whether or not a patient with DF symptoms went to a medical facility immediately. The maximum score for the Practices portion was 8 and ranged from 0–8 points. The Cronbach’s alpha coefficient of DF Prevention Practices ranged from 0.70 to 0.74 indicating an acceptable level of internal consistency [29, 30]. We calculated the mean percentage score by dividing each of the KAP mean scores by their highest possible score and, multiplied them by 100, so as to be able to compare our results with previous findings.

### Independent variables

Independent variables used for the study analyses were based on previous studies [11, 31, 32] and independent variable included were gender, age category, employment status, income class, length of stay in hospital, cost of stay in hospital in Taka which had the following options in the survey (<500, 500–3000, 3001–5500, 5501–8000, 8001–10500, 10501–13000, 13,001–15500, 15501–18000, 18001–20,500 and >20500). However, for statistical analysis purpose, the cost of stay in hospital in Taka was categorized into (≥3000 Taka and 3001 Taka) since 54.5% of the participants reported ≥3000 Taka. Other independent variables included: years resident in Dhaka, use of mosquito net (likely to be ineffective at preventing dengue which is transmitted by day-biting Aedes mosquitoes), regular cleaning of drainage system and regular visit to doctors.

### Statistical analysis

Preliminary analyses involved frequency counts of all selected independent variables. This was followed by independent t-tests to determine if the KAP scores differed by the two city-corporations (DNCC and DSCC). This was followed by a preliminary analysis on KAP scores that showed that the distribution of KAP scores was skewed towards right. KAP scores were then considered as count variable with non-negative integer values, and therefore Poisson regression was used in the analysis. Multiple Poisson regression was used to determine the adjusted estimate and their 95% confidence intervals (CIs). These were done for each district separately and for both districts in a pooled analysis to identify common factors influencing DF related KAP in Dakar City. All statistical analyses were conducted using STATA/MP Version.14.1 (StataCorp, College Station, Texas, USA). In our analysis, we checked for homogeneity of variance and multicollinearity including Variance Inflation Factors (VIF) using linear regression because collinearity is a property of the independent variables and has nothing to do with the type of regression and the VIF < 4 was considered suitable [33]. Poisson coefficients (Tables 2 and 3) and their 95% confidence intervals (CIs) were obtained from the adjusted regression model and were used to measure the factors associated with the KAP scores in two city-corporations.

## Results

**Fig 2:**
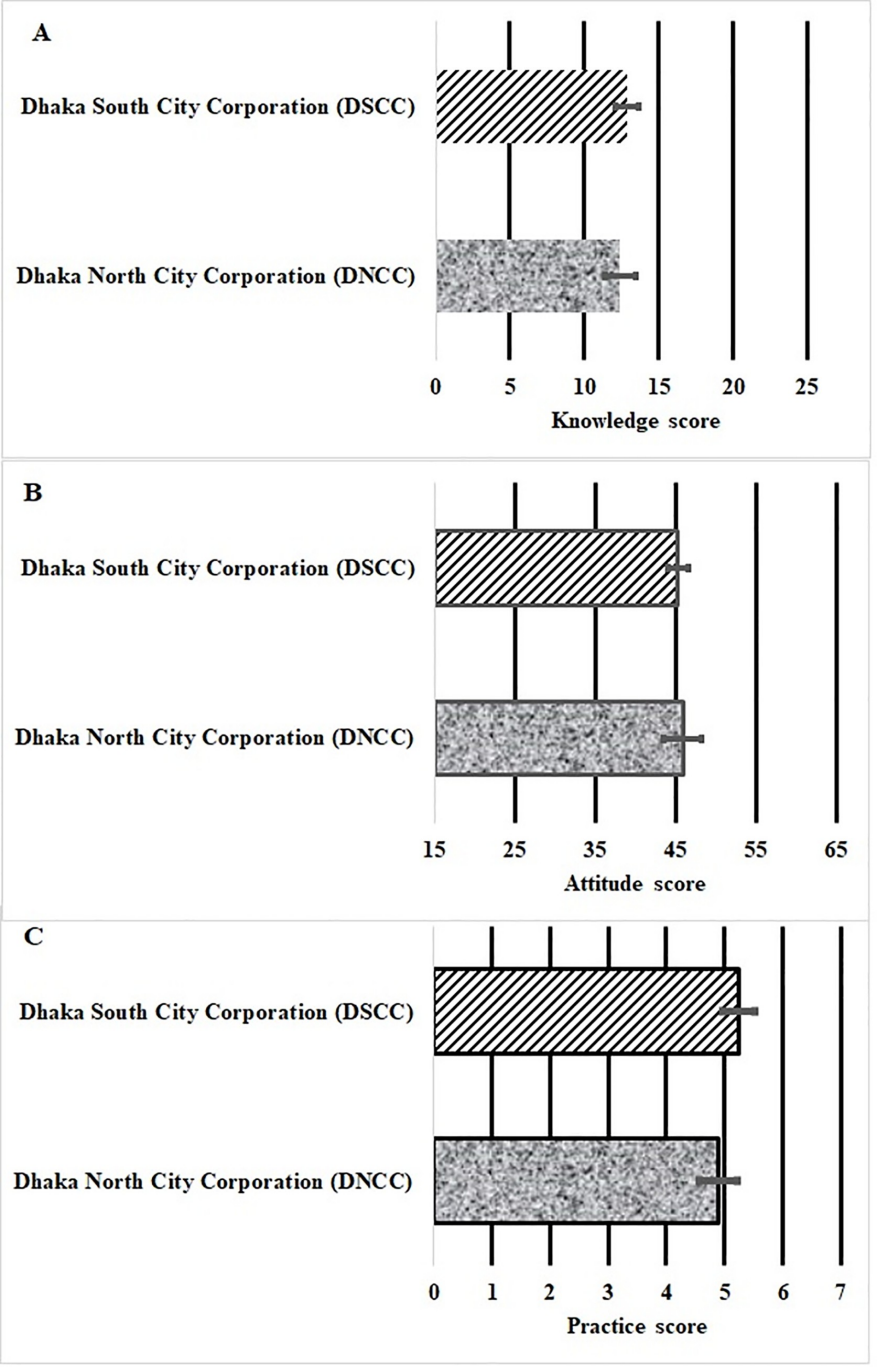
The average scores for (A) knowledge, (B) attitude (B) and (C) practice towards dengue in both DNCC and SNCC Dhaka.

Table 1 illustrates the demographic characteristics of respondents from the DNCC and DSCC. Many of the respondents were aged 20-40years (44.2%), half were females, most were employed (65.3%) and were middle to high-income earners (86%). About 62.4% of the respondents lived in the South of Dhaka. Many respondents did not protect themselves from mosquitoes (60%), and a majority (88.5%) failed to maintain good hygiene by not cleaning their drainage systems. About two thirds (71%) of the respondents reported frequent visits to a doctor and almost everyone had spent seven or less nights in hospital. The average scores for the respondents’ knowledge (A), attitude (B) and practice (C) towards dengue in DNCC and SNCC Dhaka (Fig 2) were not significantly different between DNCC and SNCC (unpaired t-test: p = 0.2268, 0.7006 and 0.062 respectively). Overall, the pooled averages (±SD) for both cities were 12.0±4.9, 45.5±9.2, and 5.1±1.7 for knowledge, attitude and practice respectively.

**Table 1:** Descriptive statistics of the sociodemographic characteristics of respondents from Bangladesh.

Characteristic	DNCC, n = 91 (37.6%)	DSCC, n = 151 (62.4%)	Total, n = 242 (100%)
**Gender**			
Male	66 (72.5)	77 (51.0)	143 (59.09)
Female	25 (27.5)	74 (49.0)	99 (40.9)
**Age category**			
<20 years	35 (38.5)	40 (26.5)	75 (31.0)
20–40 years	35 (38.5)	72 (47.7)	107 (44.2)
40+ years	21 (23.1)	39 (25.8)	60 (24.8)
**Employment status**			
Unemployed	45 (49.5)	39 (25.8)	84 (34.7)
Employed	46 (50.5)	112 (74.2)	158 (65.3)
**Income Class**			
Lower Class	33 (36.3)	20 (13.3)	53 (21.9)
Middle/Higher Class	58 (63.7)	131 (86.8)	189 (78.1)
**Length of stay in hospital**			
Below 7 Days	81 (89.0)	145 (96.0)	226 (93.4)
7 or more days	10 (11.0)	6 (4.0)	16 (6.6)
**Total cost of stay in hospital in Taka**			
≤3000	71 (78.0)	61 (40.4)	132 (54.5)
3000 or more	20 (22.0)	90 (59.6)	110 (45.5)
**Years resident in Dhaka**			
Non resident	24 (26.4)	57 (37.8)	81 (33.5)
≤10 years	36 (39.6)	41 (27.1)	77 (31.8)
10+years	31 (34.1)	53 (35.1)	84 (34.7)
**Use of Mosquito net**			
Yes	53 (58.2)	44 (29.1)	97 (40.1)
No	38 (41.8)	107 (70.9)	145 (59.9)
**Regular cleaning of drainage system**			
Yes	3 (3.3)	37 (24.5)	40 (16.5)
No	88 (96.7)	114 (75.5)	202 (83.5)
**Regular visit to Doctors**			
Yes	51 (56.0)	120 (79.5)	171 (70.7)
No	40 (44.0)	31 (20.5)	71 (29.3)

Dhaka North City Corporation (DNCC); Dhaka South City Corporation (DSCC).

Table 2 illustrates the adjusted odds for factors associated with knowledge, attitude and practice scores in both the north and south of Dhaka. For example, positive odds ratio for knowledge indicate higher scores for knowledge and generally more knowledge about dengue fever than in the reference group. After adjusting for potential confounding variables, respondents with up to 10 years of residency in Dhaka and those that failed to protect themselves from mosquitoes using mosquito nets, had lower odds of Dengue-related knowledge compared to those not resident in Dhaka and respondents that protected themselves from mosquitoes. The use of mosquito net was significantly associated with good attitude towards dengue but the association between income class and attitude towards dengue only approached significance (coefficient (β) = 0.10, 95%CI -0.998,0.20; p = 0.058) among respondents in DNCC. Respondents who spent seven or more days in hospital had lower attitude scores compared with those who spent fewer days in hospital.

**Table 2:** Adjusted coefficients and 95% confidence intervals of multivariate analysis for factors associated with knowledge, attitude and practice towards dengue in Dhaka North City Corporation (DNCC), Bangladesh.

	Knowledge score		Attitude score		Practice score	
Characteristics	Coefficient	95% CI	Coefficient	95% CI	Coefficient	95% CI
**Gender**						
Male	Reference		Reference		Reference	
Female	-0.11	-0.29, 0.07	-0.02	-0.10, 0.07	-0.02	-0.29, 0.26
**Age category**						
< 20 years	Reference		Reference		Reference	
20–40 years	0.02	-0.22, 0.27	0.02	-0.11, 0.14	-0.04	-0.43, 0.35
40+ years	0.04	-0.22, 0.30	0.05	-0.09, 0.19	0.02	-0.41, 0.45
**Employment status**						
Unemployed	Reference		Reference		Reference	
Employed	-0.02	-0.20, 0.17	-0.02	-0.12, 0.07	0.04	-0.26, 0.34
**Income Class**						
Lower Class	Reference		Reference		Reference	
Middle/Higher Class	0.02	-0.17, 0.20	0.10	-0.00, 0.20	0.04	-0.27, 0.35
**Length of stay in hospital**						
Below 7 Days	Reference		Reference		Reference	
7 or more days	-0.14	-0.45, 0.17	**-0.18**	**-0.34**, **-0.02**	0.10	-0.37, 0.57
**Total cost of stay in hospital in Taka as per minimum wage**						
≤3000	Reference		Reference		Reference	
3000 or more	-0.07	-0.28, 0.15	0.09	-0.02, 0.20	0.12	-0.22, 0.45
**Years resident in Dhaka**						
non resident	Reference		Reference		Reference	
≤10 years	-0.10	-0.29, 0.09	-0.08	-0.18, 0.02	0.14	-0.18, 0.46
10+years	**-0.23**	**-0.43**, **-0.03**	-0.08	-0.19, 0.02	0.21	-0.12, 0.54
**Use of Mosquito net**						
Yes	Reference		Reference		Reference	
No	-0.23	**-0.37**, **-0.09**	**-0.16**	**-0.24**, **-0.09**	0.11	-0.12, 0.35
**Regular cleaning of drainage system**						
Yes	Reference		Reference		Reference	
No	0.05	-0.36, 0.45	0.01	-0.19, 0.21	-0.13	-0.77, 0.50
**Regular visit to Doctors**						
Yes	Reference		Reference		Reference	
No	-0.14	-0.30, 0.02	-0.07	-0.15, 0.02	0.13	-0.11, 0.38
**Knowledge Score**	-	-	**-0.01**	**-0.02**, **-0.01**	0.01	-0.01, 0.03

Bolded are confidence intervals that do not include ‘0’ representing statistical significance.

Table 3 illustrates the factors associated with dengue-related knowledge, attitude and practice, among respondents in DSCC. After adjusting for potential cofounders, respondents’ age was the only factor associated with attitude towards dengue in DSCC, such that older respondents (aged 40 years and above) had a poorer attitude (adjusted coefficient 0.13, 95%CI 0.03, 0.23) towards dengue compared to younger people (aged 20 years and below). Knowledge scores were negatively related to attitude towards dengue (adjusted coefficient -0.01, 95%CI -0.02, -0.01) among DNCC respondents, but showed marginal positive relationship with practice towards dengue (coefficient 0.02, 95%CI 0–0.03) among respondents in DSCC. No other variable showed significant association with the outcome measures.

**Table 3:** Adjusted coefficients and 95% confidence intervals of multivariate analysis for factors associated with knowledge, attitude and practice towards dengue in Dhaka South City Corporation (DSCC).

	Knowledge score		Attitude score		Practice score	
Characteristics	Coefficient	95% CI	Coefficient	95% CI	Coefficient	95% CI
**Gender**						
Male	Reference		Reference		Reference	
Female	-0.10	-0.20, 0.09	0.04	-0.02, 0.09	0.01	-0.16, 0.17
**Age Category**						
< 20 years	Reference		Reference		Reference	
20–40 years	0.01	-0.18, 0.19	0.04	-0.06, 0.13	-0.05	-0.34, 0.23
40+ years	0.11	-0.08, 0.30	**0.13**	**0.03**, **0.23**	-0.04	-0.33, 0.26
**Employment status**						
Unemployed	Reference		Reference		Reference	
Employed	0.00	-0.18, 0.18	-0.09	-0.18, 0.01	0.18	-0.10, 0.47
**Income Class**						
Lower Class	Reference		Reference		Reference	
Middle/Higher Class	**-0.19**	**-0.32**, **-0.06**	-0.01	-0.09, 0.07	0.02	-0.20, 0.24
**Length of stay in hospital**						
Below 7 Days	Reference		Reference		Reference	
7 or more days	0.02	-0.24, 0.28	-0.02	-0.16, 0.12	0.07	-0.34, 0.49
**Total cost of stay in hospital in Taka**						
≤3000	Reference		Reference		Reference	
3000 or more	-0.01	-0.12, 0.09	-0.01	-0.07, 0.04	0.03	-0.14, 0.20
**Years resident in Dhaka**						
Non resident	Reference		Reference		Reference	
≤10 years	-0.08	-0.21, 0.05	0.01	-0.06, 0.08	-0.03	-0.23, 0.18
10+years	-0.04	-0.16, 0.08	0.02	-0.05, 0.08	0.02	-0.18, 0.21
**Use of Mosquito net**						
Yes	Reference		Reference		Reference	
No	0.02	-0.09, 0.13	-0.03	-0.09, 0.03	-0.04	-0.22, 0.14
**Regular cleaning of drainage system**						
Yes	Reference		Reference		Reference	
No	-0.10	0.21, -0.00	-0.01	-0.07, 0.05	0.07	-0.11, 0.24
**regularly visit to Doctors**						
Yes	Reference		Reference		Reference	
No	0.01	-0.12, 0.15	-0.02	-0.09, 0.05	-0.04	-0.25, 0.17
**Knowledge Scores**	-	-	0	-0.01, 0.00	**0.02**	**0.00**, **0.03**

Bolded are confidence intervals that do not include ‘0’ representing statistical significance.

In Table 4, the adjusted coefficients for the association between the variables and knowledge, attitude and practice scores are presented for the pooled corporations (DNCC and DSCC combined). The table shows that higher income earners in Dhaka and those who did not clean their drainage system regularly had lower dengue related knowledge compared with lower income earners and respondents who regularly cleaned their drainage system, following adjustment for all potential cofounders. Whereas respondents who failed to use mosquito nets while sleeping had poor attitude towards dengue, good practice towards dengue was marginally associated with dengue related knowledge (adjusted coefficient 0.01, 95% CI **0.00, 0.02**) among the respondents in this study.

**Table 4:** Adjusted coefficients and 95% Confidence Intervals (CI) of multivariate analysis for factors associated with knowledge, attitude and practice towards dengue in Dhaka, Bangladesh.

	Knowledge score		Attitude score		Practice score	
Characteristics	Coefficient	95% CI	Coefficient	95% CI	Coefficient	95% CI
**Gender**						
Male	Reference		Reference		Reference	
Female	-0.08	-0.16, 0.00	0.02	-0.02, 0.06	0.02	-0.11, 0.15
**Age Category**						
< 20 years	Reference		Reference		Reference	
20–40 years	-0.02	-0.15, 0.12	-0.02	-0.09, 0.01	0.00	-0.21, 0.21
40+ years	0.03	-0.10, 0.16	0.05	-0.02, 0.12’	0.03	-0.18, 0.24
**Employment status**						
Unemployed	Reference		Reference		Reference	
Employed	0.04	-0.08, 0.16	-0.02	-0.08, 0.04	0.10	-0.10, 0.29
**Income Class**						
Lower Class	Reference		Reference		Reference	
Middle/Higher Class	**-0.10**	**-0.19, -0.01**	0.04	-0.01, 0.10	0.04	-0.12, 0.20
**Length of stay in hospital**						
Below 7 Days	Reference		Reference		Reference	
7 or more days	-0.11	-0.28, 0.06	-0.06	-0.14, 0.03	0.10	-0.15, 0.35
**Total length of stay in hospital**					
≤3000	Reference		Reference		Reference	
3000 or more	-0.01	-0.09, 0.07	0.00	-0.05, 0.04	0.05	-0.08, 0.18
**Years resident in Dhaka**						
non resident	Reference		Reference		Reference	
≤10 years	-0.04	-0.13, 0.06	0.01	-0.04, 0.06	-0.03	-0.17, 0.12
10+years	-0.06	-0.15, 0.04	0.00	-0.05, 0.05	0.05	-0.10, 0.20
**Use of Mosquito net**						
Yes	Reference		Reference		Reference	
No	-0.08	-0.16, 0.01	**-0.07**	**-0.12, -0.03**	0.02	-0.11, 0.15
**Regular cleaning of drainage system**						
Yes	Reference		Reference		Reference	
No	**-0.11**	**-0.21, -0.02**	0.00	-0.06, 0.05	0.05	-0.11, 0.21
**Regular visit to Doctors**						
Yes	Reference		Reference		Reference	
No	-0.09	-0.18, 0.01	-0.03	-0.08, 0.02	0.01	-0.13, 0.15
**Knowledge Scores**			0.00	-0.01, 0.00	**0.01**	**0.00, 0.02**

**Bolded** are confidence intervals that do not include ‘0’ representing statistical significance. Data for Dhaka South City Corporation (DSCC) and Dhaka North City Corporation (DNCC) were pooled.

## Discussion

This study examined KAP regarding dengue among the residents of Dhaka via the two city corporations, Dhaka North City Corporation (DNCC) and Dhaka South City Corporation (DSCC). Our study showed that more than half of the study population were knowledgeable about dengue, possess an appropriate and acceptable attitude towards the disease, and engage in practices towards its prevention. However, the factors associated with KAP regarding dengue varied between both city corporations; with duration of residency and use of mosquito nets found to be correlated with knowledge in the north while income class and age were predictors of knowledge and attitude in the south. In the pooled analysis (combining both cities), knowledge of dengue was a significant predictor of good practice towards dengue fever among the respondents.

We found that the overall mean percentage scores of 52%, 69.2% and 71.4% for KAP was higher than a hospital-based study that examined KAP on dengue fever among paediatric and adult in-patients in Metro Manila, Philippines [34]. This suggests that people in Dhaka were generally more knowledgeable, concerned, and had better practices towards dengue than in the comparison study [34]. The higher mean percentage scores reported in this study could be attributed to the fact that participants examined in this study may have experienced an episode of dengue which could change their perceptions and attitude towards the disease.

As has been previously established [35], the use of mosquito nets in the evenings does not add any value in the prevention of dengue as the virus is transmitted by day-biting mosquitoes. Despite the absence of a direct link between bed net usage and dengue prevention, it is possible that people who use bed nets for the prevention of other mosquito-borne disease (e.g. malaria) may be more aware of the risk posed by mosquito bites and may be more actively preventing mosquito bites through the day. It is not clear why this association was only observed in Dhaka North area.

Most respondents were aware that the mosquitoes that transmit dengue, *Aedes aegypti* and *Aedes albopictus*, require stagnant water to propagate and could lay eggs even in clean water containers suggesting an increase in knowledge of dengue transmission when compared to the results from earlier studies in Dhaka [31, 36]. This upward trend in knowledge may be due to the intensified educative campaigns from health authorities owing to the continued outbreaks and exponential increase in the number of dengue cases in the last six years [9]. For example, in 2014, there were about 375 reported dengue cases with no recorded fatalities in Bangladesh. In contrast, more than 87,953 cases and 81 deaths were reported in 2019 [9]. Even though the knowledge of the mosquito’s habit and life-cycle may be lacking [37], people were more likely to have heard about dengue or know someone who has been diagnosed with the disease.

Despite a high level of knowledge about dengue and required preventative measures, there were no corresponding intentional drive to intentionally combat the spread of dengue in either locality (DNCC or DSCC). This survey further revealed that many respondents were cognizant that indiscriminate dumping of plastic containers and bottles could lead to the creation of mosquito larval habitats. However, less than a quarter of the participants inappropriately disposed unused water-holding containers. These containers are potential breeding sites for mosquitoes that may transmit infections, including dengue [38]. The significant association between knowledge and attitude did not yield positive practices that could prevent the spread of dengue. Even though most respondents claimed to appreciate the potential severity of dengue and were inclined to seek medical attention in a hospital during an infection, they did not engage in activities that could get rid of mosquitoes and larvae in their environment. For example, only 16.5% of participants cleaned their drainage system regularly. While this is consistent with many KAP surveys where high levels of awareness of dengue did not expressly translate to practices that can deter disease transmission [39–42], some studies showed that good knowledge manifests as acceptable attitude and satisfactory practices to prevent the disease [32, 43].

The exact factor that inhibits the translation of knowledge of dengue to adequate preventive practices remains elusive but may stem from a common belief that it is the not their obligation or that it is the government’s sole responsibility to deploy measures to control mosquito populations and curtail the spread of dengue in the locality [44, 45]. This sense of entitlement which has also been reported elsewhere [46] should be discouraged as it may hinder individual efforts, and promote a nonchalant attitude to vector control measures despite the attendant health benefits [39, 47]. It is expedient that public campaigns against the spread of dengue should emphasize the importance of prevention practices including getting rid of mosquito breeding sites. Small community groups should also channel their efforts at enforcing practical measures like appropriate disposal of containers and bottles that are potential breeding sites for disease vectors. It is the responsibility of every member of the community to ensure their houses or flats are free of breeding sites to protect their own households and those of their neighbours.

The duration of residency in Dhaka North had statistically significant association with knowledge of dengue. However, patients that have lived in Dhaka for more than ten years had lower odds of being able to identify clinical symptoms, describe means of transmission and understand means of preventing propagation of larvae and mosquitoes in this study. It is likely that Dhaka being a capital city with a greater government presence is exposed to more frequent dengue awareness and prevention campaigns compared to outside areas.

In Dhaka South, income class was associated with good knowledge. Some studies on dengue have demonstrated that a direct relationship exists between income class and knowledge [48, 49], and attitude [49, 50]. In this study, we found that respondents in the middle and higher socioeconomic class had lower dengue related knowledge, as they were less likely to correctly identify clinical symptoms of dengue fever and its means of transmission and prevention relative to those in the lower income class. This could be because people with better economic standing have better health facilities [32, 51] and may have little or no experience of the disease compared with those of lower socioeconomic class. As in many other diseases, the ability to identify symptoms of dengue will enable diagnosis and treatment and improve clinical outcomes in dengue as in many other diseases. It is therefore imperative that quality access to health information cut across the socioeconomic strata in any given community.

This study found a significant association between age and attitude of the patients towards dengue in Dhaka South. Participants aged 40 years and above were less likely to appreciate the severity of dengue and the necessity of hospitalization due to dengue than people aged 20 years or less. In contrast to our findings, another study from Bangladesh [44], found that those aged 45–60 years were more likely to report positive attitude towards undertaking precautionary measures to prevent dengue than those aged less than 25 years. Here, it is noteworthy that a present limitation in KAP surveys is the fluid concept of attitude despite scoring systems. While this study defined attitude as the feeling about the severe levels of dengue and the necessity of hospitalization due to dengue; elsewhere [44], attitude refers to the feelings of the community members towards dengue prevention and control. Therefore, it is not surprising that the varying indices in different studies resulted in different measurement outcomes. Nevertheless, it is reasonable that perspectives on dengue transmission are dynamic among young people in Dhaka given the drastic changes in both dengue infections and related fatalities in the last six years.

In this study, the duration of hospital stay was found to be a predictor for attitude towards dengue. Responding residents who were hospitalised for seven or more days reported better attitude towards dengue as compared to people who spent less than seven days in the hospital. This could be explained by the fact that there is no substitute for firsthand experiences since longer hospital stay and increased perceived vulnerability are important drivers of positive attitude and dengue prevention practices [52]. Acknowledging the severity of dengue and the importance of seeking medical attention in a hospital at the onset of symptoms should be deemed necessary and not optional; as it could reduce the duration of hospital stay.

Overall, the significant association between the different outcome variables of KAP are in agreement with previous studies [49, 53]. The association between knowledge and practice is not static with some previous studies reporting significant positive associations [37, 40, 48, 54], and others reported no significant associations [55]. A successful and sustainable approach to translate knowledge into appropriate prevention practices must be one that is community-oriented and people-friendly. Central to the improvement of attitude and practice is behavioural change which though requires basic knowledge but is fuelled by the willingness to change the status quo. Awareness campaign messages should seek to establish the relationship between positive outcomes of good practices in the community and improvements in the health of individuals. As a way of improving the practices of communities, practical approaches in health education should be tailored to discourage negative community habits like indiscriminate refuse disposal and poor maintenance of drains.

Another limitation of this study is that the responses to the questionnaire may not be reflective of actual attitude and practices given that it was a self-reported survey and the respondents may seek to provide socially acceptable responses even though the survey was anonymized. First, the cross-sectional study design limits the causality of the relationship between KAP and associated factors. Second, this study cannot be generalisable to the wider Dhaka population because participants were recruited from the Hospital. Thus, this may limit the generalizability of the study. Third, the reported KAP score might be higher than the general population because the study took place in urban hospitals where participants may have experienced an episode of dengue that may change the participants perceptions and attitude towards the dengue fever. Fourth, this study lacks an appropriate or equivalent comparison group and so does not demonstrate cause. Finally, it is possible the patients will have provided responses that they viewed as favourable to the interviewers, but the use of trained interviewers who could recognise such bias will minimise this bias.

## Conclusion

This study found that fever patients in Dhaka had knowledge of dengue, with regards to its clinical symptoms, means of transmission and prevention strategies, and there was a relatively positive attitude towards the severity of the disease and the need for hospitalization. There is need to institute practical and comprehensive public health measures that incorporates behavioural impact assessment at the grassroots level and channelled towards the translation of awareness into preventive practices, since mere awareness is not sufficient on its own. Closing the gap in quality access to accurate health information is key and should be directed equally across all strata of society, especially in developing countries like Bangladesh. Access to information will inform real and tangible intervention strategies for the prevention of dengue. Practical, family-oriented and community-based health education campaigns should be tailored to discourage negative community habits like indiscriminate refuse disposal and lack of drain maintenances and inspire healthy family practices that mitigate the risk of dengue spread.

## Supporting information

S1 Table A study on the cause and current situation the spread of dengue (a mosquito-borne tropical disease caused by the dengue virus) in Bangladesh. (DOCX)
