# Supplementary material for: Dengue in Dhaka, Bangladesh: Hospital-based cross-sectional KAP assessment at Dhaka North and Dhaka South City Corporation area
Source: PLoS One. 2021 Mar 30;16(3):e0249135. doi: 10.1371/journal.pone.0249135 (PMC8009423; doi:10.1371/journal.pone.0249135)
Supplement: S1 Table — (DOCX) [file pone.0249135.s001.docx]

**S1 Table.**

**A study on the cause and current situation the spread of dengue (a mosquito-borne tropical disease caused by the dengue virus) in Bangladesh**

**Basic Questions**

| Name of the patient/person |  | | Gender: | Male Female |
| --- | --- | --- | --- | --- |
| Age in years |  | | Hospital / Clinic Name |  |
| Working status | Employed  Unemployed | |  |  |
| District name | DSCC  DNCC | | A permanent resident of Dhaka | Yes No |
| On average, how much do you spend in hospital for Dengue treatment during any single admission  Financial status / monthly income | Choose any option  Less than 500 Taka  500 - 3000  3001 - 5500  5501 - 8000  8001 - 10500  10501 - 13000  13,001 - 15500  15501 - 18000  18001 - 20,500  More than 20500  Lower class /Middle class / upper class | | How long have you been in hospital at any one time due to Dengue | …………………  DAYS |
| Dengue test lunar rate |  | |  |  |
| Phone Number |  | | E-mail |  |
| Blood group |  | Formerly in dengue (NS1-Positive) You’ve been attacked? | | Yes No |

**Knowledge and behavior in relation to the hygiene and health of persons with dengue**

Note: Tick mark your opinion /answer in the blank space next to the question

1. How long have you lived in this city?

Days . . . . . . months, . . . . . . . . year

1. Did you know, Dengue eggs can live in clean water? Yes No
2. Are the drainage systems/canals /drains/rivers regularly cleaned in your area?

Yes No

1. Do you use mosquito nets regularly during sleep?

Yes No

**Knowledge on dengue.**

1. Sign and symptoms of dengue **(Multiple answer acceptable**)
2. Continuous fever
3. Skin rash
4. Muscle pain
5. Nausea/vomiting
6. Headache
7. Small bleeding at gum and nose
8. Diarrhea
9. Where are the potential breeding spots inside house? **(Multiple answer acceptable)**
10. Pot liner
11. Kitchen cutlery tray
12. Toilet flush/tank
13. Cistern
14. Refrigerator tray
15. Where are the potential breeding spots outside house? **(Multiple answer acceptable)**
16. Clogged gutter/drain
17. Water storage container, jar
18. Unclosed cans and bottles
19. Solid waste that can stored water
20. Old tires that can stored water
21. How to prevent breeding spot. **(Multiple answer acceptable)**
22. Allow fogging inside the house
23. Managed waste and recyclables properly
24. Used mosquito aerosol spray
25. Install mosquito nets at house windows
26. Prevent stagnant water
27. Clear the clogged water every 3 days
28. Always closed containers that can stored water
29. Eliminate mosquito breeding spots weekly
30. Install mosquito nets at house windows
31. Use mosquito nets at the time (day time) of sleeping

**Practice on Aedes and dengue prevention.**

1. Are you throw waste in tied plastic trash and tightly closed the trash bin?
2. Yes b) No
3. Are you Ensure water containers was tightly closed each time after used?
4. Yes b) No
5. Are you destroy damaged equipment that can stored water?
6. Yes b) No
7. Are you clean water inside container once a week?
8. Yes b) No
9. Are you check for mosquito breeding spot outside the house?
10. Yes b) No
11. Are you check for mosquito breeding spot inside the house?
12. Yes b) No
13. Are you use mosquito aerosol/spray every day?
14. Yes b) No
15. Are you use mosquito net during daytime sleeping?
16. Yes b) No

**Attitude scoring of the respondents**

1. I Feel that dengue fever is very dangerous
2. Strongly agree
3. Agree
4. Neutral
5. Disagree
6. Strongly disagree
7. I do not think that dengue fever can cause death
8. Strongly agree
9. Agree
10. Neutral
11. Disagree
12. Strongly disagree
13. Dengue fever can occur without the presence of skin rashes
14. Strongly agree
15. Agree
16. Neutral
17. Disagree
18. Strongly disagree
19. I get worried when I have continuous fever
20. Strongly agree
21. Agree
22. Neutral
23. Disagree
24. Strongly disagree
25. Even though there are various medical facilities for treatment, I am still afraid of dengue fever
26. Strongly agree
27. Agree
28. Neutral
29. Disagree
30. Strongly disagree
31. I get very worried when get bitten by a mosquito
32. Strongly agree
33. Agree
34. Neutral
35. Disagree
36. Strongly disagree
37. Having a standing of water that is unnecessary outside my house does not bother me
38. Strongly agree
39. Agree
40. Neutral
41. Disagree
42. Strongly disagree
43. You can be infected with dengue fever
44. Strongly agree
45. Agree
46. Neutral
47. Disagree
48. Strongly disagree
49. If you are healthy, you will not be infected with dengue fever
50. Strongly agree
51. Agree
52. Neutral
53. Disagree
54. Strongly disagree
55. I am often bitten by mosquitoes but never fall sick. So I cannot be infected with dengue fever
56. Strongly agree
57. Agree
58. Neutral
59. Disagree
60. Strongly disagree
61. Do you believe that dengue fever could be prevented and controlled?
62. Strongly agree
63. Agree
64. Neutral
65. Disagree
66. Strongly disagree
67. Do you think that eliminating the breeding site of the dengue-causing mosquito is complicated and time consuming?
68. Strongly agree
69. Agree
70. Neutral
71. Disagree
72. Strongly disagree
73. Do you think that you have an important role in dengue fever prevention?
74. Strongly agree
75. Agree
76. Neutral
77. Disagree
78. Strongly disagree
79. Do you think that dengue is a major problem for your population?
80. Strongly agree
81. Agree
82. Neutral
83. Disagree
84. Strongly disagree
85. Do you think that dengue is difficult to detect?
86. Strongly agree
87. Agree
88. Neutral
89. Disagree
90. Strongly disagree
91. Do you think that the environment is conducive to disease transmission?
92. Strongly agree
93. Agree
94. Neutral
95. Disagree
96. Strongly disagree
97. Do you think that dengue has a high morbidity?
98. Strongly agree
99. Agree
100. Neutral
101. Disagree
102. Strongly disagree
103. Do you think dengue is considered an alarming diagnosis?
104. Strongly agree
105. Agree
106. Neutral
107. Disagree
108. Strongly disagree
109. Do you feel like dengue decreases economic productivity?
110. Strongly agree
111. Agree
112. Neutral
113. Disagree
114. Strongly disagree
